# Bacterial Muramyl Dipeptide (MDP) Restricts Human Cytomegalovirus Replication via an IFN-β-Dependent Pathway

**DOI:** 10.1038/srep20295

**Published:** 2016-02-02

**Authors:** Arun Kapoor, Yi-Hsin Fan, Ravit Arav-Boger

**Affiliations:** 1 Department of Pediatrics, Division of Infectious Diseases, Johns Hopkins University School of Medicine, Baltimore, Maryland 21287, USA

## Abstract

We recently reported that induction of NOD2 by human Cytomegalovirus (HCMV) resulted in virus inhibition and upregulation of antiviral and inflammatory cytokines. Here we investigated the effects of muramyl dipeptide (MDP), a bacterial cell wall component that activates NOD2, on HCMV replication and antiviral responses. HCMV infection of human foreskin fibroblasts induced NOD2, the downstream receptor-interacting serine/threonine-protein kinase 2 (RIPK2), resulting in phosphorylation of TANK-binding kinase 1 (TBK1) and interferon regulatory factor 3 (IRF3). MDP treatment following infection at low multiplicity (MOI = 0.1 PFU/cell) inhibited HCMV in a dose-dependent manner and further induced phosphorylation of TBK1, IRF3 and expression of IFN-β. None of these effects of MDP were observed following infection at multiplicity of 1. In infected NOD2 knocked-down cells MDP did not induce IFN-β, irrespective of MOI. Treatment with MDP before infection also inhibited HCMV, an effect augmented with treatment duration. Treatment with an IFN-β receptor blocking antibody or knockdown of IFN-β significantly attenuated the inhibitory effect of MDP on HCMV. MDP treatment before or after infection with herpesvirus 1 did not inhibit its replication. Summarized, NOD2 activation exerts anti-HCMV activities predominantly via IFN-β. Since MDP is a bacterial cell wall component, ongoing microbial exposure may influence HCMV replication.

Infection with human CMV (HCMV), a member of the herpesvirus family, is common in humans. Seroprevalence rates increase with age, reaching 80–90% in individuals older than 80 years^1^. While infection in the normal host is usually asymptomatic, HCMV is a major pathogen in immunocompromised patients and in congenitally-infected newborns^2^,^3^,^4^. In these cohorts infection can be severe, persistent, recurrent, or resistant to anti-viral therapy.

Recognition of HCMV by the innate immune system can set the cell for an effective response and is counteracted by HCMV to allow for productive replication and generation of latency. Thus, uncovering the initial responses to HCMV may be critical in developing measures to prevent or inhibit virus replication. Reported pattern recognition receptors (PRRs) for HCMV have already indicated that its recognition is a complex and a multi-step process. The membrane Toll-like receptor 2 (TLR2) recognized HCMV and triggered an inflammatory cytokine production via interaction with HCMV-encoded glycoproteins B and H^5^,^6^. Several cytosolic and nuclear PRRs have been identified as sensors for herpesviruses^7^. The DNA-dependent activator of interferon (IFN)-regulatory factors (DAI), activated interferon regulatory factor 3 (IRF3) upon infection with HCMV and its constitutive overexpression inhibited virus replication^8^. IFN-inducible protein, IFI16, inhibited HCMV by blocking Sp1-mediated transcription of HCMV genes involved in viral DNA synthesis^9^. The nucleotide-binding oligomerization domain and leucine rich repeat containing receptor, NLRC5, was induced by HCMV within 24 h after infection and its knock-down impaired the upregulation of interferon alpha in response to HCMV^10^.

We recently reported that the nucleotide binding oligomerization domain 2 (NOD2), a cytoplasmic PRR and a known susceptibility marker for Crohn’s disease, was upregulated by HCMV resulting in induction of IFN-β and inhibition of HCMV^11^. NOD2 recognizes a muramyl dipeptide (MDP) moiety, present on most types of bacterial peptidoglycans. Although NOD1 and NOD2 are well-established intracellular sensors of bacteria^12^,^13^,^14^,^15^,^16^,^17^,^18^,^19^, recent studies indicate that RNA viruses can activate specialized signaling downstream of NOD2^11^,^20^,^21^. The induction or activation of NOD2 by different pathogens may result in upregulation of a pathogen-specific immune responses.

Since induction of NOD2 by HCMV resulted in consequential virus inhibition^11^, and since MDP is known to bind to and activate NOD2^22^, we investigated the effects of MDP on HCMV replication and the pathway through which MDP exerts anti-HCMV activities. Our results show that MDP has dose-dependent anti-HCMV activities which are augmented during exposure time and are IFN-β dependent. These data suggest a new hypothesis that ongoing microbial exposure and potentially the microbiome generates an environment that may suppress HCMV.

## Materials and Methods

### Reagents and chemicals

MDP was obtained from Invivogen (San Diego, CA) and dissolved in endotoxin free water. A stock of 10 mg/mL was prepared and stored at −20 °C. Unless otherwise specified MDP was used at a concentration of 10 μg/mL as per previous studies for NOD2 activation. Ganciclovir (Sigma Aldrich, St. Louis, MO) was used at 5 μM.

### Cell culture and viruses

All infection experiments were performed with human foreskin fibroblasts, passage 12–16 (ATCC, CRL-2088™). Cells were grown in Dulbecco’s Modified Eagle’s Medium (DMEM) containing 10% fetal bovine serum (FBS) (Gibco, Carlsbad, CA) in a 5% CO_2_ incubator at 37 °C. The generation of NOD2-knockdown cells (shNOD2) and control cells (GIPZ) was reported^11^. One day prior to infection or treatment, 8 × 10^4^ cells were seeded into each well of 12-well tissue culture plates. Multiplicities of infection were 0.1 or 1 in wild type cells, or 0.5 and 3 in lentivirus transduced cells, to achieve similar degree of infectivity between wild-type and transduced cells. The Towne HCMV strain was obtained from ATCC (VR-977). The pp28-luciferase Towne which expresses luciferase under the control of the pp28 late promoter has been described, and correlates well with plaque reduction^23^. Luciferase activity was measured at 72 or 96 hours post infection (hpi) using the Glomax-Multi + Detection System (Promega, Madison, WI). In second cycle infection assays cell free supernatants from pp28-luciferase infected cells were collected 4 days post infection (5–10% of total volume/well), and used for infection of fresh cells seeded into 12-well plates. Cell lysates were collected after 72 h for luciferase activity. Supernatants from the first cycle were also used to measure virus titer and plaques were counted at day 8 post infection. A purified preparation of the pp28-luciferase Towne strain was prepared by ultracentrifugation using sucrose gradient^24^. The HCMV TB40 strain was obtained from ATCC (VR-1578). Clinical isolates of HCMV were obtained from the microbiology laboratory at Johns Hopkins Hospital with no identifiers that could be linked to a patient. Human Herpesvirus 1, (HSV1)-luciferase (KOS/Dlux/oriS) was provided by Dr. David Leib, Dartmouth Medical School.

### Plaque assay

A plaque reduction assay was performed to determine HCMV inhibition by MDP. Cells were seeded into 12-well plates (2 × 10^5^ cells/well) and infected with Towne HCMV at approximately 100 plaques/well. After 90 minutes, media were aspirated, and DMEM containing 0.5% carboxymethyl-cellulose (CMC), 4% fetal bovine serum (FBS), and MDP were added into duplicate wells. In pretreatment experiments the same procedure was used, but cells were first treated with MDP for 72 h followed by infection. After incubation at 37 °C for 8 days (Towne) or 10 days (TB40) the overlay was removed and plaques were counted after crystal violet staining. A plaque assay was also performed to determine the effect of MDP on HSV1 replication. The same procedure was performed as for HCMV other than adsorption time of 60 minutes after infection with 50 plaques/well and counting of plaques after 48 h.

### Add-on assay

Cells were infected with HCMV Towne (MOI 0.1). At 0, 8, 24, 48 and 72 hpi, the medium was replaced with fresh medium containing MDP. A Western blot for HCMV proteins IE1/2, pp65, and β-actin was performed at 96 hpi. A plaque assay was performed as described above to determine the effect of timing of MDP addition on HCMV replication.

### Cellular toxicity

MTT assay (Sigma-Aldrich) was performed to rule out any cytotoxicity induced by MDP. Non-infected or HCMV-infected cells were treated with MDP for 96 h, and 20 μl/well of MTT [3-(4, 5-Dimethyl-2-thiazolyl)-2, 5-diphenyl-2 H-tetrazolim bromide, 5 mg/mL in phosphate buffered saline (PBS)] was added to each well. After shaking plates at 150 rpm for 5 minutes the plates were incubated at 37 °C for 3 hours. Conversion of yellow solution to dark blue formazan by mitochondrial dehydrogenases of living cells was quantified by measuring absorbance at 560 nm.

### Lentivirus-mediated knockdown of IFN-β

Human GIPZ lentiviral shRNAmir constructs (Open Biosystems, Huntsville, AL) were used for IFN-β knockdown. Five clones (V2LHS_238693, V3LHS_355340, V3LHS_355342, V3LHS_355343, V3LHS_355344) targeting different regions of IFN-β mRNA were tested and the clone (V3LHS_355343) with the best knockdown efficiency was selected for further experiments. GIPZ non-targeting control plasmid was used to rule out non-specific effects of shRNAmir constructs. Individual shRNAmir constructs were packaged using lentivirus as described previously^25^. Briefly, 21 μg of gag/pol, 7 μg of vesicular stomatitis virus glycoprotein, and 7 μg of shRNAmir plasmids were transfected into HEK293 cells using calcium phosphate method. After 48 h the packaged lentivirus particles were concentrated from the medium. The supernatant was filtered and centrifuged at 1750 g for 30 min at 4 °C in Amicon Ultra (Ultracel 100k, Millipore). After centrifugation, 2 ml of cold PBS was added and the tubes were centrifuged again for 20 min at 4 °C. The concentrated virus was stored at −80 °C until used. Lentivirus particles containing shRNAmir were transduced into Human Foreskin Fibroblasts. 0.5 × 10^6^ cells were plated onto T-25 flask and 40 μl of concentrated virus and Polybrene (final concentration, 8 μg/mL) were added to the cells, and incubated for 4 h. Following transduction puromycin (2 μg/mL) was added to select for stably transduced cells.

### RNA isolation and real-time quantitative reverse transcriptase (qRT)-PCR

Total RNA was isolated from cells using RNAeasy Mini kit (Qiagen, Georgetown, MD) according to manufacturer’s instructions. RevertAid first strand cDNA synthesis kit (Fermentas life sciences, Cromwell Park, MD) was used to synthesize first strand cDNA from total RNA using oligo-dT primers. Negative reverse-transcriptase (-RT) reactions were included to ensure the specificity of qRT-PCR reactions. Synthesis of first strand cDNA from mRNA template was carried out at 42 °C for 1 h. Quantitative RT-PCR was performed using specific primers and SYBR green (Fermentas life science) with two-step cycling protocol (95 °C for 15 s, 60 °C for 1 min). All reactions were performed in triplicates and GAPDH was used as internal control. The expression level of the tested genes was normalized to the expression of GAPDH. The primer sequences were: NOD2-Forward, 5′-GCCACGGTGAAAGCGAAT-3′, NOD2-Reverse, 5′-GGAAGCGAGACTGAGCAGACA-3′, IFN-β-Forward 5′-GATTCATCTAGCACTGGCTGG-3′, IFN-β-Reverse, 5′-CTTCAGGTAATGCAGAATCC-3′, IL8-Forward, 5′-TGCAGCTCTGTGTGAAGGTGCAGT-3′, IL8-Reverse, 5′-CAGTGTGGTCCACTCTCAATCACTC-3′, RIG-I-Forward, 5′- ATCCCAACCGATATCATTTCTGATC-3-, RIG-I-Reverse, 5′- TTCCACCAATTTCTCTGCACCTGC-3, MDA5-Forward, 5′- GGCACCATGGGAAGTGATT-3, MDA5-Reverse, 5′- GATGATGATATTCTTCCCTTCCA-3, GAPDH-Forward 5′-TTGGTATCGTGGAAGGACTC-3′ and GAPDH-Reverse, 5′- ACAGTCTTCTGGGTGGCAGT-3′.

### SDS-polyacrylamide gel electrophoresis and immunoblot analysis

Cell lysates containing equivalent amount of proteins were mixed with an equal volume of sample buffer (125 mM Tris-HCL, pH 6.8, 4% SDS, 20% glycerol and 5% *β*-mercaptoethanol) and boiled at 100 °C for 10 min. Denatured proteins were resolved in Tris-glycine polyacrylamide gels (10–12%) and transferred to polyvinylidine difluoride (PVDF) membranes (Bio-Rad Laboratories, Hercules, CA) by electroblotting. Membranes were incubated in blocking solution [5% w/v non-fat dry milk and 0.1% Tween-20 in PBS (PBST)] for 1 h, washed with PBST, and incubated with antibody at 4 °C overnight. Membranes were washed with PBST and incubated with horseradish peroxidase-conjugated secondary antibodies in PBST for 1 h at room temperature. Following washing with PBST, protein bands were visualized by chemiluminescence using SuperSignal West Dura and Pico reagents (Pierce Chemical, Rockford, IL). Antibodies for HCMV proteins were used at 1:2000 and included: mouse monoclonal anti-HCMV IE1 & IE2 (MAB810, Millipore, Billerica, MA), mouse monoclonal anti-HCMV UL83 (pp65, Vector Laboratories Inc., Burlingame, CA), and mouse monoclonal anti-pp52 (UL44, Santa Cruz Biotechnology, Santa Cruz, CA). Rabbit polyclonal anti human-NOD2 antibody (1:2000), rabbit polyclonal anti-RIPK2 (1:5000), mouse monoclonal anti-NF-κB (p65, 1:1000), rabbit polyclonal anti-IRF3 (1:1000) and mouse monoclonal anti-IRF7 (1:1000) were from Santa Cruz. Rabbit monoclonal anti-Histone H3 (1:2000), rabbit monoclonal anti-pIκBα (1:2000), mouse monoclonal anti-IκBα (1:2000), rabbit monoclonal anti-pTBK1 (Ser172, 1:1000), and rabbit monoclonal anti-TBK1 (1:1000) were from Cell Signaling Technology, Beverly, MA. Horseradish peroxidase (HRP)-conjugated anti-rabbit IgG was from Cell Signaling. Horseradish peroxidase (HRP)-conjugated anti-mouse IgG was from GE Healthcare (Waukesha, WI). Mouse monoclonal anti-human β-actin 1:5000 was from Sigma. Mouse monoclonal anti-human IFNα/β receptor chain 2 antibody (EMD Millipore Corporation, CA) was used at 5 μg/mL to block the IFN-β receptor. Anti-mouse IgG (Santa Cruz) was used as control in the IFN-β receptor blocking experiment.

### Preparation of cytoplasmic and nuclear extracts

Cytoplasmic and nuclear fractions were isolated from HCMV-infected or mock-infected cells at 24 hpi as previously reported^11^. Briefly, cells were washed twice with ice-cold PBS and resuspended on ice for 15 min in buffer A containing 10 mM HEPES (pH 7.9), 10 mM KCl, 0.1 mM EDTA, 1 mM dithiotheitol (DTT), protease and phosphatase inhibitors. Cells were then lysed by adding 0.1% NP40 and cytosolic supernatants were obtained by centrifugation at 10,000 rpm for 30 sec. Crude nuclei were washed twice with buffer A to prevent cytoplasmic contamination, and nuclear proteins were extracted by resuspending cell pellets with buffer C containing 20 mM HEPES (pH 7.9), 400 mM NaCl, 1 mM EDTA, 1 mM DTT, protease and phosphatase inhibitors. The mixture was incubated for 15 min with vigorous shaking on rocker at 4 °C and then centrifuged at 14,000 rpm at 4 °C for 10 min to obtain the nuclear proteins. Protein concentration was determined using BCA protein assay reagent kit (Pierce Chemical, Rockford, IL).

### ELISA

Levels of secreted IFN-β in supernatants from non-infected or HCMV-infected cells were measured by IFN-β specific ELISA kit (PBL Assay Science, Piscataway, NJ).

### Immunofluorescence staining for HCMV IE1/2

GIPZ control and shIFN-β cells were seeded into 96-well plate (1 × 10^4^ cells/well) and pretreated with MDP for 72 h followed by infection with a clinical isolate of HCMV at MOI 1. Twenty-four h after infection, cells were fixed with methanol/acetone (1:1) at −20 °C for 10 min. Cells were then blocked with 7.5% BSA for 30 min. and incubated for 1 h with anti-human CMV IE1&2 antibody (MAB810) (1:500 in 0.5% BSA). After 3 washes with PBS, cells were incubated with FITC-anti mouse antibody (Sigma, 1:500, in 0.5% BSA) for 1 h. Then cells were washed 3 times with PBS and counterstained with propidium iodide (Invitrogen) for nuclear staining. Images were taken using Nikon Eclipse TS100 microscope.

### Densitometry and statistical analysis

Quantitative analysis of proteins detected by immunoblotting was performed using ImageJ 1.48v software (NIH) by determining band intensities relative to β-actin, as reported by others^26^. Statistical analysis was performed using one-way ANOVA comparison between different groups with significance value set at *p* < 0.05.

## Results

### MDP inhibits HCMV replication at low MOI

**Figure 1:**
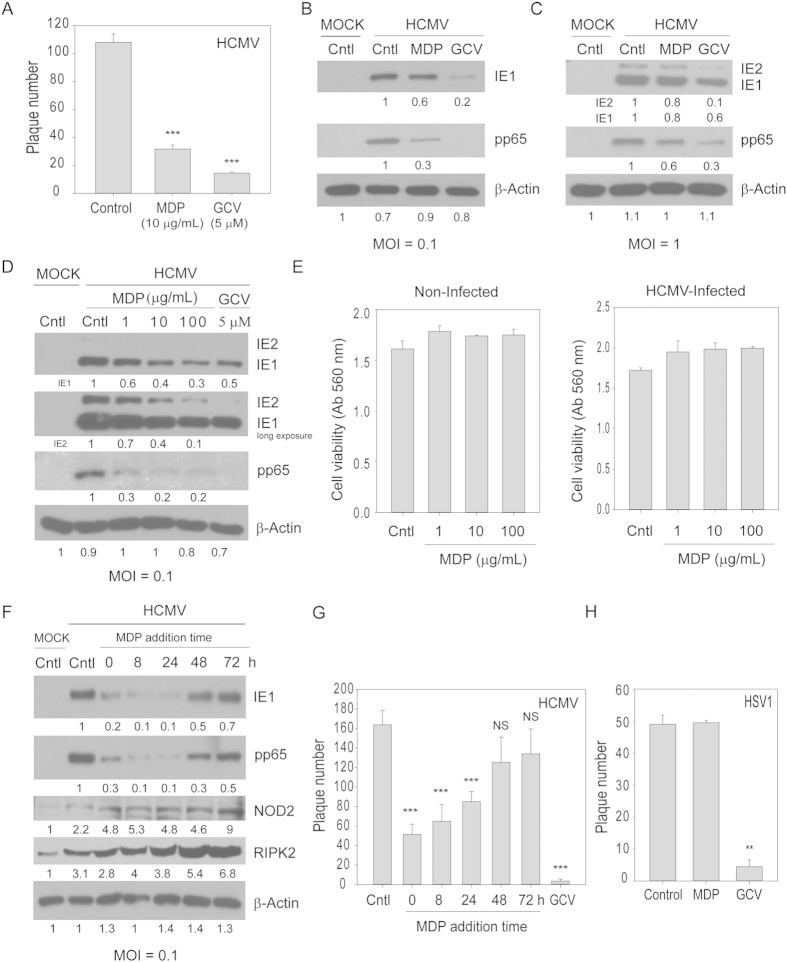
MDP treatment inhibits HCMV replication at low MOI. (**A**) Cells were infected with HCMV-Towne (100 PFU/well) and treated with MDP. The number of plaques, counted after 8 days, are average data from two independent experiments. (**B**,**C**) Cells were infected with HCMV-Towne (MOI 0.1 and 1) and treated with MDP. The expression level of indicated proteins was determined at 4 days post infection. (**D**) Dose response curve of MDP. Cells were infected (MOI 0.1) and treated with indicated concentrations of MDP or GCV. The expression of IE1/2, pp65, and β-actin was determined after 4 days. (**E**) Non-infected or HCMV-infected cells were treated with MDP for 96 h and cell viability was determined using an MTT assay. (**F**,**G**) Add-on assay of MDP (10 μg/mL). Cells were infected and MDP added at the indicated time points. The expression of HCMV proteins, NOD2 and RIPK2 was measured at 96 hpi (**F**) and a plaque reduction assay was performed at day 8 post infection (**G**). (**H**) Cells were infected with HSV1 (100 PFU/well) and treated with MDP. The number of plaques, counted after 2 days, are average data from two independent experiments. The numbers below the blots show relative band intensity compared to β-actin. *** denotes *p* < 0.001, and **p < 0.01. NS, non-significant.

To determine the effect of MDP on virus replication, human foreskin fibroblasts were infected with the HCMV Towne strain at 100 PFU/well and treated with MDP or ganciclovir (GCV). MDP reduced plaque number (Fig. 1A) and expression of HCMV proteins (Fig. 1B). The effect of MDP on the expression of IE1/2 and pp65 was MOI dependent (Fig. 1B,C). At 96 hpi pp65 expression was decreased by approximately 75% and 40% at MOI 0.1 and 1, respectively. At MOI 0.1 MDP suppressed HCMV replication in a dose-dependent manner (Fig. 1D), an effect that was not secondary to cellular toxicity, since an MTT assay performed during the same time point revealed no toxicity in non-infected or HCMV-infected cells at any of the concentrations used (Fig. 1E). Addition of MDP at different times after infection revealed that IE1 and pp65 expression was significantly reduced when MDP was added after 8 h and before 48 hpi, suggesting its inhibitory effects occurred mainly at an early stage of virus replication (Fig. 1F). Similarly, MDP significantly reduced the number of plaques when added before 48 hpi (Fig. 1G). We previously reported that induction of NOD2 by HCMV was relatively low before 24 hpi and that UV-inactivated HCMV could not induce NOD2^11^. However, in that study we did not measure the timing of HCMV inhibition with MDP. The add-on assay (Fig. 1F) improves our understanding of the effects of MDP on HCMV suppression, indicating that the most effective time of MDP activity is before 48 hpi. Although when added to infected cells after 48 h, MDP could still induce NOD2 and RIPK2 expression (Fig. 1F), HCMV replication (by protein expression and plaque reduction) was not inhibited, indicating virus replication took over signaling for its benefit. HSV1 was not inhibited by MDP (Fig. 1H), suggesting a specificity of the NOD2 pathway for HCMV inhibition.

### MDP and HCMV cooperate in the induction of NOD2 and IFN-β

**Figure 2:**
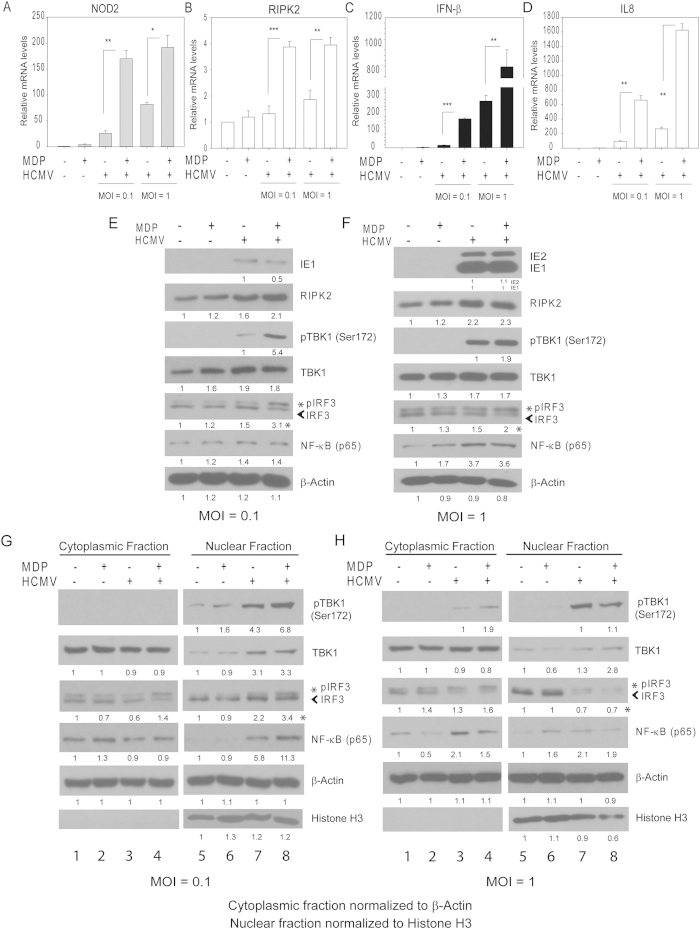
Effect of MDP on expression of NOD2, IFN-β, and IL8 in HCMV-infected cells. (**A**–**D**) Non-infected or HCMV-infected cells (MOI 0.1, and 1 PFU/cell) were non-treated or treated with MDP after infection. Levels of NOD2, RIPK2, IFN-β, and IL8 mRNA were measured by qRT-PCR at 36 hpi. The relative mRNA expression was compared to non-infected non-treated cells, and represents the induction level for each RNA tested. Data shown are mean ± SD from triplicate wells of a representative experiment of two independent experiments (*p < 0.05, **p < 0.01, ***p < 0.001, one-way ANOVA test). (**E**,**F**) The expression of proteins downstream of NOD2 in cells infected with MOI 0.1 (**D**) or 1 (**E**) was determined by Western blot at 36 hpi. (**G**,**H**) Expression of proteins downstream of NOD2 was measured in the cytoplasmic and nuclear fractions at 36 hpi. The asterisk (*) represents pIRF3 in the quantified blots. Western blot data are from a representative experiment of three independent experiments

Several signaling pathways are activated downstream of NOD2: The NF-κB is the classic pathway which promotes transcription of pro-inflammatory cytokines (such as IL-8). Amongst alternative pathways is type I IFN. Different pathogens preferentially activate a signaling downstream of NOD2^19^,^27^,^28^. Since MDP inhibited HCMV replication, we investigated its effects on IFN-β and IL8 expression in infected cells. NOD2, RIPK2, IFN-β and IL8 mRNA was measured at 36 hpi (Fig. 2A–D), a time point selected based on our previous study showing significant NOD2 induction at 24 hpi and afterwards. The combination of HCMV and MDP resulted in enhanced induction of NOD2 mRNA, compared to the induction achieved with HCMV infection or MDP alone (Fig. 2A). MDP treatment resulted in 4-fold induction of NOD2 mRNA in non-infected cells. After infection at multiplicity of 0.1 or 1 MDP induced NOD2 by 6.5- and 2.5-fold, respectively (Fig. 2A). There was no significant induction of RIPK2 mRNA after HCMV infection. RIPK2 mRNA was induced by 3- and 2-fold with MDP treatment after MOI of 0.1 and 1, respectively, suggesting its activity may not be regulated at the transcriptional level (Fig. 2B). IFN-β expression was induced 4-fold by MDP, 15-fold by HCMV (MOI 0.1) and 300-fold by HCMV (MOI 1.0) compared to its expression in non-infected cells. MDP treatment after low MOI (0.1) resulted in significant induction of IFN-β mRNA compared to infection-only (170- vs 15-fold), an effect that was modest at MOI 1 (800- vs 300-fold induction, Fig. 2C). These results demonstrate an enhanced effect of MDP and HCMV in inducing IFN-β expression at low MOI.

The mRNA expression of the pro-inflammatory cytokine, IL-8, was measured. IL8 expression was induced 3-fold by MDP, 90-fold by HCMV (MOI 0.1) and 260-fold by HCMV (MOI 1). MDP treatment after infection further increased (6–7 fold) IL8 mRNA compared to MDP or HCMV alone, but in contrast to IFN-β induction this change was irrespective of MOI (Fig. 2D).

### MDP treatment following HCMV infection further induces NOD2 downstream signaling

Upon detection of MDP, NOD2 binds to RIPK2 via CARD-CARD homophilic interactions, a step required for downstream signaling to proceed^27^. RIPK2 is a critical kinase downstream of NOD2, since in RIPK2-deficient cells, NOD2 signaling is abolished. We investigated the effect of MDP on expression of NOD2 downstream signaling proteins in HCMV-infected cells at 36 hpi (Fig. 2E,F). Infection at multiplicity of 0.1 caused a 2.5-fold increase in RIPK2 expression (Fig. 2E, lanes 1&3) and further increase was observed in infected MDP-treated cells (lanes 3&4). The phosphorylation of TANK-binding kinase (TBK1) and IRF3 was further increased in MDP-treated HCMV-infected cells compared to their levels in infection alone (Fig. 2E, lanes 3&4). The increased phosphorylation of TBK1 and IRF3 was also observed in the nuclear fraction (Fig. 2G). At higher MOI, MDP treatment failed to further induce RIPK2, pTBK1 or pIRF3 (Fig. 2F -total cell lysates, Fig. 2H - cytoplasmic and nuclear fractions). MDP treatment following infection with HSV1 (MOI 0.1) showed no further induction of IRF3 or NF-κB (Supplementary material, Fig. S1).

As expected, NF-κB (p65) relocalized to the nucleus after HCMV infection (Fig. 2G). At MOI 0.1, MDP increased nuclear localization of NF-κB (Fig. 2G, lanes 7&8), an effect that was not observed at MOI 1 (Fig. 2F, lane 7&8). These results suggest that that the downstream effects of MDP on TBK1 and IRF3 phosphorylation are MOI-dependent and at higher multiplicity HCMV could overcome the MDP-induced changes in the respective signaling proteins. The effects of MDP in HCMV-infected cells were also tested at an earlier time point (4 hpi). There was only 2-fold increase in IFN-β and IL8 mRNA, and no changes were observed in the expression of signaling proteins downstream of NOD2, irrespective of MOI (Supplementary material, Fig. S2).

### Activation of the IFN-β pathway by MDP at low MOI is NOD2-dependent

**Figure 3:**
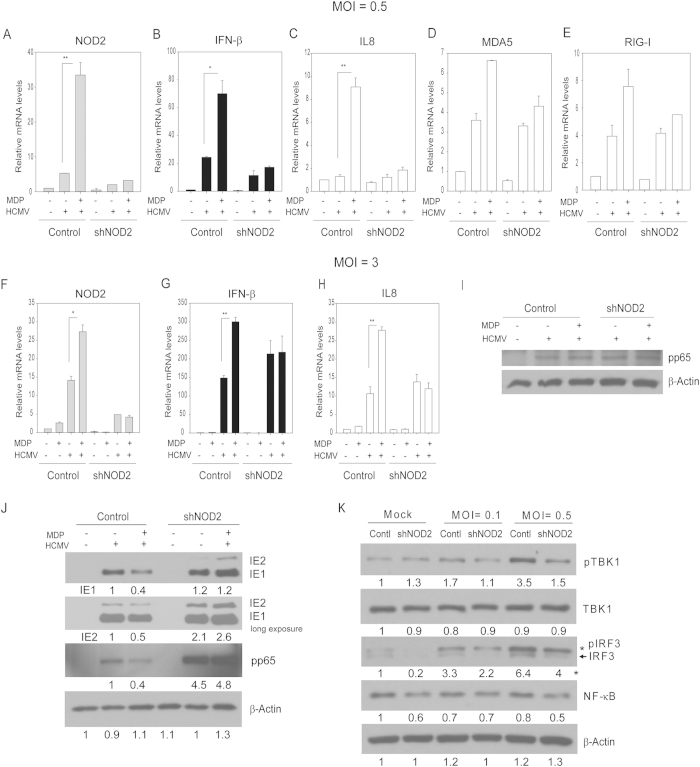
Induction of NOD2, IFN-β and IL8 mRNA in HCMV-infected MDP-treated cells is NOD2-dependent. (**A–E**) Control (GIPZ) or shNOD2 cells were mock- or HCMV-infected (MOI 0.5) and were either non-treated or treated with MDP for 48 h. NOD2, IFN-β, IL8, MDA5, and RIG-I mRNA was measured by qRT-PCR. The depicted mRNA expression experiments represent mean ± SD from triplicate wells of a representative experiment. (**F**–**H**) Control (GIPZ) or shNOD2 cells were mock- or HCMV-infected (MOI 3), and either non-treated or treated with MDP for 36 h. NOD2, IFN-β and IL8 mRNA was measured by qRT-PCR. Data shown are mean ± SD from triplicate wells of a representative experiment (*p < 0.05, **p < 0.01, one-way ANOVA test). (**I**) For virus entry assay the control or shNOD2 cells were infected with HCMV Towne (MOI = 3) for 2 h at 37 °C, washed with citric acid buffer (pH = 3) to strip off virus particles adhered to cell surface, and pp65 was detected by Western blot. (**J**) Control (GIPZ) or shNOD2 cells were mock- or HCMV-infected (MOI 0.1) and were either non-treated or treated with MDP for 96 h. Levels of IE1/2, pp65, and β-Actin were determined by Western blot. (**K**) Control (GIPZ) or shNOD2 cells were mock- or HCMV-infected (MOI 0.1, and 0.5) for 24 h. Levels of NF-κB, IRF3, pTBK1, TBK1, and β-Actin was determined by Western blot. The western blot data are representative of two independent experiments.

Since MDP directly binds to and activates NOD2^22^, we investigated whether induction of NOD2, IFN-β, and IL8 transcripts by MDP in infected cells was NOD2-dependent. HCMV-infected NOD2-knockdown (shNOD2) and GIPZ control cells were treated with MDP (Fig. 3A-C). Similar to non-transduced cells (Fig. 2), MDP treatment after infection further induced NOD2, IFN-β and IL8 mRNAs in control (GIPZ) infected cells. There was no such induction after MDP treatment in infected shNOD2 cells irrespective of MOI, confirming the requirement and specificity of NOD2 for MDP activities during infection (Fig. 3A,D). In addition, mRNA induction of MDA5 or RIG-I following HCMV infection was similar between control and NOD2 knockdown cells, and MDP treatment resulted in less than 2-fold induction of these mRNA, indicating again its specific effects through NOD2 (Fig. 3D,E). At higher MOI, NOD2 expression was also significantly reduced in the shNOD2 cells. IFN-β and IL8 were induced by infection but no further upregulation by MDP was observed in the shNOD2 cells (Fig. 3F,H). These results suggest that MDP-induced changes are through NOD2, and at higher MOI NOD2-independent pathways upregulate IFN-β and IL8. The observed changes were not secondary to differences in infection efficiency between the cell lines, since a Western blot for pp65 at 2 hpi showed similar virus entry (Fig. 3I). Western blot performed at 96 hpi in NOD2 knockdown and control cells, revealed decreased IE1/2, and pp65 expression after MDP treatment only in the control cells, but not in the NOD2 knockdown cells (Fig. 3J). In addition, levels of NF-ĸB, pTBK1 and IRF3 were mildly reduced in the NOD2 knockdown cells. Taken together (Figs 1, 2, 3), MDP treatment after low MOI could enhance antiviral responses in a NOD2-dependent manner via activation of TBK1 and IRF3, resulting in decreased virus replication.

### Prolonged exposure to MDP before infection inhibits HCMV replication

**Figure 4:**
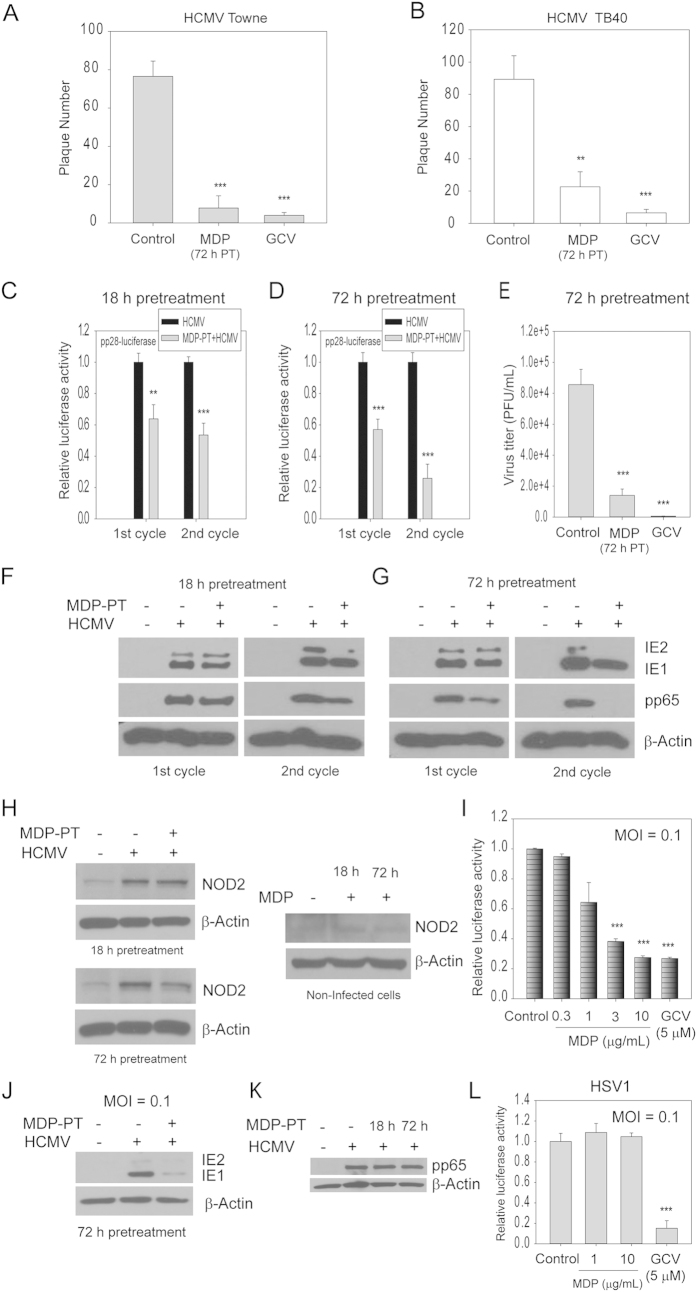
MDP pretreatment inhibits HCMV replication. (**A**,**B**) Cells were pretreated with MDP for 72 h followed by infection with Towne or TB40 (100 PFU/well). GCV was used after infection. The number of plaques, counted after 8 or 10 days, represent average data from two independent experiments. (**C**,**D**) Cells were pretreated with MDP for 18 or 72 h followed by infection with pp28 luciferase HCMV (MOI 1). Luciferase activity was measured at 96 hpi (1st cycle). Supernatants were collected at 96 hpi for a 2nd cycle infection and luciferase activity was measured after 72hpi. Data are mean ± SD from two independent experiments (**p < 0.01, ***p < 0.001, one-way ANOVA test). (**E**) Supernatants from pp28-luciferase HCMV-infected cells were used to infect fresh cells and plaques were counted after 8 days. (**F**,**G**) Cells were pretreated with MDP for 18 or 72 h and then infected with HCMV (MOI 1) for 96 h. Supernatants were collected at 96 hpi for 2nd cycle infection and cell lysates were collected at 72 hpi. The expression of IE1/2, pp65 and β-actin from the 1st and 2nd cycle was measured by Western blot. (**H**) Cells were non-treated or pretreated with MDP for 18 or 72 h and then infected with HCMV (MOI 1) for 96 h. NOD2 expression was determined by Western blot. NOD2 expression was also determined in non-infected cells treated with MDP for 18 or 72 h. (**I**) Dose-response of MDP pretreatment at MOI 0.1. Cells were pretreated with MDP for 72 h at the indicated concentrations, followed by infection. GCV was added after infection. Luciferase activity was measured at 96 hpi. (**J**) Cells were non-treated or pretreated with MDP for 72 h, then infected with HCMV (MOI 0.1) for 96 h. Expression of indicated proteins was determined by Western blot. (**K**) HCMV entry into MDP pre-treated or non-pretreated cells was determined by Western blot for pp65 at 2 hpi. (**L**) Cells were non-treated or pretreated with MDP for 72 h, then infected with HSV1-luciferase for 24 h. GCV was added after infection as a positive control for HSV1 inhibition.

To begin understanding a potential ongoing role for MDP derived from bacterial pathogens in HCMV suppression we tested whether MDP exposure *prior* to infection could generate an antiviral environment. Cells were pretreated with MDP for 72 h followed by infection with Towne or TB40 strains of HCMV (100 PFU/well), and plaques were counted after 8 and 10 days, respectively. In MDP-pretreated cells the plaque number was reduced by approximately 90% and 70% for Towne and TB40, respectively (Fig. 4A,B). The effect of duration of MDP exposure on HCMV replication was tested by pretreatment for 18 or 72 h followed by infection with the pp28 luciferase-recombinant HCMV-Towne (MOI 1). Luciferase activity was modestly reduced at 96 hpi following 18 or 72 h of MDP pretreatment (Fig. 4C,D). Supernatants from infected cells (non-pretreated or MDP-pretreated) were collected after 4 days (completion of first cycle) and used for infection of fresh cells (second cycle). Luciferase activity after second cycle infection was reduced by approximately 45% and 75% in cells pretreated with MDP for 18 or 72 h, respectively (Fig. 4C,D). Supernatants from the first cycle were also used for virus titration in fresh cells and a plaque assay performed at 8 days post infection revealed a 6-fold reduction in virus titer in MDP-pretreated cells (Fig. 4E). MDP pretreatment resulted in reduced expression of HCMV IE1/2, and pp65 (Fig. 4F,G). The longer MDP pretreatment (72 h), the more significant was the reduction of IE2 and pp65 expression in second cycle infection. Similar effects of MDP on HCMV replication were observed when a purified virus preparation was used (Supplementary material, Fig. S3). NOD2 expression was induced in HCMV-infected cells at 96 hpi, and MDP pretreatment did not further increase NOD2 expression after infection (Fig. 4H). Treatment of non-infected cells for 72 h resulted in minor induction of NOD2 protein level (Fig. 4H), suggesting the duration of MDP treatment may affect NOD2 levels. Compared to MOI 1 (Fig. 4D), at MOI 0.1, MDP pretreatment reduced luciferase activity by 80% already during the first cycle (Fig. 4I), and resulted in significant reduction of IE1/2 expression (Fig. 4J), suggesting that similar to the MOI dependency observed when MDP was added after infection, MDP pretreatment was more efficient in virus inhibition at lower MOI. These effects were not secondary to differences in HCMV uptake, since a Western blot for pp65 at 2 hpi of MDP-pretreated or non-pretreated cells showed similar levels (Fig. 4K). There was no effect on herpesvirus 1 (HSV1) replication in MDP pretreated cells (Fig. 4L), indicating specific effects of MDP on inhibition of HCMV.

### MDP pretreatment augments IFN-β response during HCMV infection

**Figure 5:**
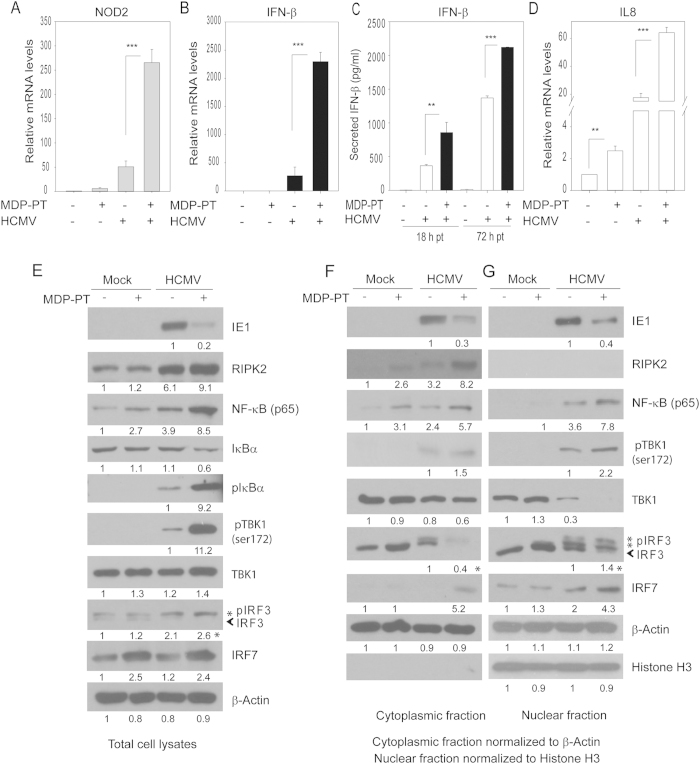
MDP-pretreatment elicits strong IFN-β response after HCMV infection. (**A**,**B**) Cells were pretreated with MDP for 72 h, and infected with HCMV (MOI 1) for 24 h. NOD2 and IFN-β mRNA was quantified by qRT-PCR. (**C**) Secreted IFN-β was measured by ELISA (**D**) Cells pretreated with MDP for 72 h, were infected with HCMV (MOI of 1) for 24 h. IL8 mRNA was quantified by qRT-PCR. Data represent mean ± SD from triplicate wells of a representative experiment of two independent experiments (**p < 0.01, ***p < 0.001, one-way ANOVA test). (**E**–**G**) The expression level of NOD2-downstream signaling proteins, those regulating IFN-β expression, and viral proteins were determined in total cell lysates (**E**), cytoplasmic (**F**) and nuclear fractions (**G**). β-actin was used as loading control, Histone H3 was used as control for nuclear proteins. The asterisk (*) represents pIRF3 in the quantified blots. Western blot data are from a representative experiment of three independent experiments.

Since MDP pretreatment inhibited HCMV replication (Fig. 4), and its addition after infection induced the phosphorylation of TBK, IRF3 and IFN-β expression (Fig. 2C,E,F), we hypothesized that the antiviral effects of MDP pretreatment were mediated through IFN-β. After pretreatment with MDP for 72 h infection was carried out with HCMV Towne (MOI 1) for 24 h. NOD2, IFN-β and IL8 transcripts were quantified by qRT-PCR. Infection in MDP-pretreated cells induced NOD2 mRNA significantly more than infection only (265- vs 50-fold, Fig. 5A). A parallel experiment in non-infected cells (72 h MDP treatment followed by washing with PBS and incubation of the cells in 4% FBS containing medium for another 24 h) resulted in only 3-fold induction of NOD2 mRNA (Fig. 5A), suggesting the effects of MDP were specifically augmented in infected cells. Similarly, infection in MDP-pretreated cells resulted in significant induction of IFN-β mRNA (Fig. 5B), and a parallel experiment in non-infected cells did not result in IFN-β induction. IFN-β levels were measured by IFN-β-specific ELISA assay in supernatants collected from MDP-pretreated cells (for 18 or 72 h) followed by infection at MOI 1 for 24 h. Secreted IFN-β increased with the duration of MDP-pretreatment (Fig. 5C, p < 0.01). MDP-pretreatment modestly and similarly induced IL8 expression in non-infected and HCMV-infected HFFs, suggesting its effects on IL8 were independent of infection (Fig. 5D), and supporting the changes in IL8 expression which were irrespective of MOI (Fig. 3C,H). These results demonstrate that MDP exposure prior to HCMV infection significantly induces IFN-β mRNA upon infection as well as extracellular secreted IFN-β. The increased IFN-β response in MDP-pretreated cells may create an unfavorable environment for HCMV replication.

### MDP pretreatment induces the classical (NF-κB) and alternative pathways (TBK1-IRF3/7) downstream of NOD2 in HCMV-infected cells

To understand the enhanced IFN-β response in MDP-pretreated cells, the expression of proteins downstream of NOD2 and their cytoplasmic/nuclear localization was determined at 24 hpi. HCMV induced RIPK2 and NF-κB expression by 6- and 4- fold, respectively (Fig. 5E, lanes 1&3), and further increase was observed with MDP pretreatment (Fig. 5E lanes 3&4). NF-κB expression is regulated by inhibitory IκB proteins, which are regulated by upstream IκB kinases (IKKs)^29^. Phosphorylation of IκB proteins results in their degradation and release of the NF-κB complex. In MDP pretreated-HCMV-infected cells IκBα expression was decreased, accompanied by significant increase in pIκBα (Fig. 5E). The changes in RIPK2 expression were observed in the cytoplasmic fraction with no evidence for nuclear relocalization. MDP pretreatment increased nuclear relocalization of NF-κB in infected cells (Fig. 5F,G). TBK1 phosphorylation was significantly induced in MDP-pretreated-infected cells (Fig. 5E) and pIRF3 was also increased. In the nuclear fraction total TBK1 expression was reduced but its phosphorylated form increased (Fig. 5G). Activated IRF3 (pIRF3) was increased in the cytoplasmic and nuclear fractions of infected cells. MDP-pretreatment followed by infection did not further increase pIRF3 (Fig. 5F), but a hyperphosphorylated form of IRF3 remained elevated in the nuclear fraction.

IRF7 expression was increased in total cell lysates after MDP pretreatment of non-infected and HCMV-infected cells (Fig. 5E). In the cytoplasmic fraction IRF7 was only detected in MDP-pretreated infected cells. Infection mildly increased nuclear IRF7 expression and a further increase was observed in MDP-pretreated HCMV-infected cells, suggesting that MDP triggered IRF7 localization and possible activation in the nuclear fraction of infected cells (Fig. 5G). As expected, at 24 hpi HCMV-IE1 expression was reduced in MDP-pretreated cells, compared to non-pretreated cells (Fig. 5E–G).

### The anti-HCMV activities of MDP require IFN-β and are significantly attenuated in IFN-β knockdown cells

**Figure 6:**
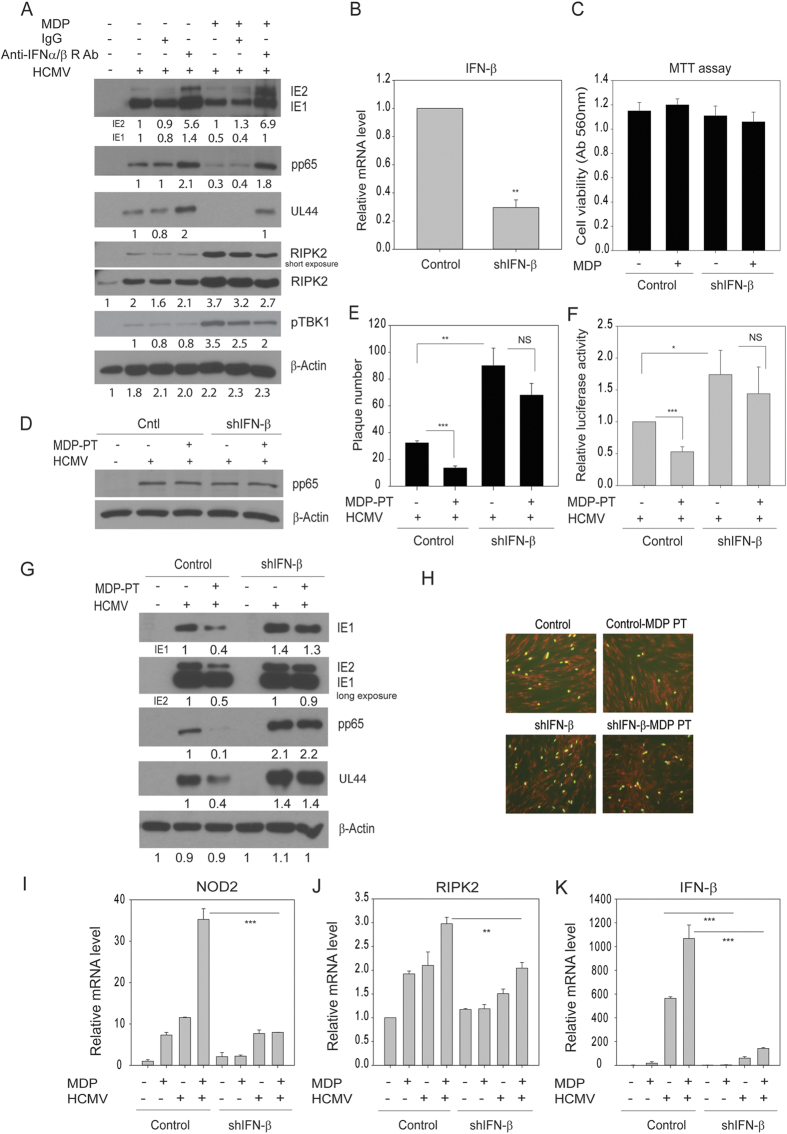
Inhibition of HCMV replication by MDP pretreatment requires IFN-β. (**A**) Cells were pretreated with MDP (72 h) followed by infection with Towne HCMV. IFN-α/β receptor blocking antibody (5 μg/mL) was added to infected cells. Expression of indicated viral and cellular proteins was measured after 4 days. Representative blots from two independent experiments are shown. (**B**) Cells were stably transduced with lentivirus expressing control (GIPZ) or shRNA against IFN-β (shIFN-β) and infected with Towne HCMV (MOI 1) for 72 h. Levels of IFN-β mRNA were measured by qRT-PCR. Data are average of duplicate values from two independent experiments. (**C**) Control or shIFN-β expressing cells were non-treated or treated with MDP and cell viability was determined after 72 h. (**D**) Virus entry into lentivirus transduced cells was determined by Western blot for pp65 following infection for 2 h and washing of cells with citric acid buffer. (**E**) Control or shIFN-β cells were non-treated or pretreated with MDP for 72 h followed by infection with HCMV Towne (100 PFU/well). The number of plaques, counted after 8 days, are average data from two independent experiments. (**F**) Control or shIFN-β cells were non-treated or pretreated with MDP for 72 h followed by infection with pp28-luciferase Towne (MOI 1) for 72 h. Luciferase activity was measured at 72 hpi. Data are average of triplicate values from three independent experiments. (**G**) Control or shIFN-β cells were non-pretreated or pretreated with MDP for 72 h and then infected with HCMV (MOI 1). Expression of IE1/2, UL44 and pp65 was determined by Western blot. Representative blots from two independent experiments are shown. (**H**) Control or shIFN-β cells were non-pretreated or pretreated with MDP for 72 h and then infected with a clinical isolate of HCMV (MOI 1). Expression of IE1/2 was determined at 24 hpi using immunofluorescence microscopy. Representative pictures from two independent experiments are shown. (**I**–**K**) Control or shIFN-β cells were mock-infected or infected with HCMV (MOI 1), and cells were untreated or treated with MDP for 24 h. Levels of IFN-β, NOD2 and RIPK2 mRNAs were measured by qRT-PCR. Data are average of triplicate values from a representative experiment of two independent experiments. *** denotes *p* < 0.001, and **-*p* < 0.01. NS, non-significant.

Since MDP pretreatment inhibited HCMV replication, and higher IFN-β response was elicited in MDP-pretreated infected cells, we tested whether the augmented NOD2-dependent IFN-β response was necessary for suppressing HCMV replication. An IFN-α/β receptor blocking antibody added to infected cells attenuated the antiviral activity of MDP (Fig. 6A), indicating that IFN-β may play an important role in the anti-HCMV activities of MDP. Levels of RIPK2 and pTBK1 were measured at 96 hpi in MDP-treated control cells (IgG) and those treated with IFN-α/β receptor antibody, showing reduction in RIPK2 and pTBK1 only in the IFN-α/β receptor antibody treated cells (Fig. 6A), suggesting the effects of MDP were mediated through IFN-β.

Human foreskin fibroblasts stably expressing shRNAs against the IFN-β gene were generated using lentiviral vectors. Five clones expressing different lentiviral shRNAs were tested and the clone resulting in the highest KD of IFN-β expression was selected for additional experiments (Fig. 6B). Equal number of control (GIPZ) and IFN-β-shRNA expressing cells (shIFN-β) were mock treated or pretreated with MDP for 72 h and after 3 days cell viability was determined by MTT assay. No toxicity or difference in cell growth was observed in MDP-pretreated control or IFN-β knockdown cells (Fig. 6C). Virus entry was similar among the different cell lines, with or without MDP pretreatment (Fig. 6D). A plaque assay was performed in control and shIFN-β cells pretreated with MDP for 72 h, followed by infection with HCMV Towne (100 PFU/well). The number of plaques, counted at day 8 post infection, was significantly decreased in MDP-pretreated control cells while in shIFN-β cells the plaque number increased. In shIFN-β cells treatment with MDP failed to restrict virus replication and the plaque number was similar between control/untreated vs MDP pretreated cells (Fig. 6E). Similarly, pretreatment with MDP for 72 h followed by infection with the pp28 luciferase-recombinant Towne strain for 72 h (MOI 1) revealed decreased luciferase activity in control cells, while in MDP-pretreated shIFN-β cells, luciferase activity was enhanced (Fig. 6F). MDP pretreatment of control cells resulted in reduced IE1/2 expression as well as significant reduction of UL44 and pp65 expression. The expression of UL44, pp65 and IE1/2 in shIFN-β cells was increased compared to control cells, and MDP pretreatment in these cells had no effect on the expression of viral proteins, demonstrating that the antiviral effects of MDP were mediated through IFN-β (Fig. 6G). An immunofluorescence staining of nuclear IE1/2 in control and shIFN-β cells, pretreated with MDP for 72 h, and infected for 24 h with a clinical isolate of HCMV showed reduced IE1/2 expression in the control cells, but not in the shIFN-β cells (Fig. 6H). Lastly, mRNA levels of NOD2, RIPK2 and IFN-β were measured in control and IFN-β knockdown cells infected and treated with MDP for 24 h. While MDP treatment induced NOD2 and IFN-β mRNA in the infected control cells, it did not have this effect in the infected IFN-β knockdown cells (Fig. 6I–K), suggesting regulation of NOD2 activities through IFN-β. There was no significant change in mRNA level of RIPK2, in agreement with Fig. 2B, again pointing to translational or post-translational modification of RIPK2 following HCMV infection, which may be modulating its downstream effects.

## Discussion

NOD2, a PRR for bacterial pathogens and a susceptibility marker for Crohn’s disease, was recently reported to recognize RNA viruses^20^. We reported on NOD2 induction by HCMV which triggered an antiviral cytokine response and suppressed virus replication, although it remains to be determined whether NOD2 acts as a true PRR for HCMV^11^,^20^. In cells overexpressing the NOD2 mutant (3020C), HCMV replication was not inhibited and IFN-β was not induced. These results prompted the current investigation of whether NOD2 activation by MDP could restrict HCMV replication, the requirement of the IFN-β pathway to HCMV suppression by MDP, and the signaling involved in NOD2 activation in infected cells.

We report here on the anti-HCMV activities of MDP, when used either after or before infection. In both cases IFN-β induction downstream of NOD2-RIPK2-TBK1 was a predominant pathway for virus inhibition. MDP activity in HCMV-infected cells was mediated through NOD2, since in NOD2 knockdown cells IFN-β was not further induced. MDP activity was dose-dependent and occurred at an early stage of HCMV replication, between 8 h and 48 hpi, after which virus replication was no longer suppressed although MDP could still induce the expression of NOD2-RIPK2. Thus, there appears to be a time-sensitive effect on signaling through NOD2 which results in HCMV inhibition (Fig. 1F). MDP treatment after low MOI (0.1) induced the IFN-β pathway downstream of NOD2, evident by increased phosphorylation of TBK1 and IRF3 (Fig. 2E). NF-κB may also contribute to the induction observed in IFN-β^30^. Neither pTBK1, pIRF3, nor NF-κB were induced in MDP-treated cells after infection at MOI 1, suggesting that NOD2-downstream signaling cannot overcome higher titer infection. Similarly, IFI16, a recently identified PRR for HCMV restricted HCMV replication at a low MOI (0.05), but not at MOI of 1 PFU/cell, although its activities were IFN-β independent^9^. Since IFI16 senses both HCMV and HSV1, and our data show that MDP did not inhibit HSV1, it is likely that CMV suppression through NOD2 and IFI16 represent independent mechanisms, both of which act at a low MOI.

Inhibition of HCMV replication with MDP pretreatment was also more efficient at low MOI, although NOD2 downstream signaling could be activated also at MOI 1, was time-dependent and increased with longer exposure. In the pretreatment condition, prior NOD2 activation was capable of overcoming a higher MOI, but in the post-treatment experiments, in which the effect MDP occurred in the setting of virus replication, it was more limited by the MOI. Following MDP pretreatment, pTBK1, a hyperphosphorylated form of IRF3 and IRF7 were increased in the nuclear fraction of infected cells, suggesting that TBK1 activation downstream of NOD2 induced antiviral response through IRF3 and 7. Activated (phosphorylated) IRF7 was reported to form heterodimers with IRF3 and promote increased expression of IFN-β^31^. To the best of our knowledge, induction of IFN-β through NOD2 has not been reported in infections with DNA viruses. There is evidence from bacterial models that IRF7 and IRF5 can be activated through NLRs^13^,^28^. In a report on *Helicobacter pylori*, RIPK2 was induced by NOD1 and activated IκB kinase ε (IKKɛ) and IRF7, followed by synthesis of type I IFN and signaling of the latter through IFN-stimulated gene factor 3 (ISGF3). *Mycobacterium tuberculosis* upregulated NOD2 - RIPK2, which activated IRF5 and induced IFNα/β^28^. Using IFN-β knockdown cells we show that the anti-HCMV activities of MDP are mediated predominantly through IFN-β. Cellular ISGs are induced upon infection with HCMV^32^,^33^. The data of IFN-β receptor blocking antibody suggest that MDP may stimulate certain ISGs via induction of IFN-β. Some of these ISGs are likely required for the anti-HCMV activity of MDP. Similar to the induction of RIPK2 with MDP treatment of HCMV- infected cells, type I IFN signaling was reported to induce RIPK2 expression and downstream signaling in macrophages with a variety of stimuli^34^. Our results show that in IFN-β knockdown cells, MDP treatment of infected cells does not induce NOD2. Although in macrophages the basal expression of NOD2 may be higher than in non-infected HFFs, the data from the two different cell types suggest that NOD2 activities may require IFN-β.

The role of NOD2 in viral recognition is just beginning to be uncovered and most information is available from studies of RNA viruses. RSV activated NOD2- mitochondrial antiviral signaling protein (MAVS) and the IFN pathway, but RIPK2 was not required for these activites^20^. RSV induced IFN-β, resulting in upregulation of NOD2. Subsequent activation by MDP induced higher proinflammatory cytokine response, suggesting that mucosal colonization with bacterial components might enhance proinflammatory cytokine responses and lead to more severe RSV disease in young children^35^. Similarly, infection of murine macrophages with murine norovirus-1 (MNV1) induced NOD1 and NOD2 in an IFN-β-dependent manner, and subsequent bacterial infection enhanced activation of NOD1/2, mediated by type I IFNs^34^. Crosstalk between type I IFNs and NOD1/NOD2 signaling has been suggested to promote bacterial recognition, resulting in harmful effects in virally-infected host. These studies differ from ours in that MDP was only used after (not before) virus infection and we investigated the effects of MDP both prior to and after HCMV infection. In addition, activation of innate/adaptive immune responses against these RNA viruses are expected to differ from those activated by HCMV, since the RNA viruses will eventually be cleared while HCMV must employ mechanisms to counteract NOD2 activity, achieve productive replication and latency.

Recent studies are beginning to identify a role for the gut virome in disease processes. Using stool samples from patients with inflammatory bowel disease, changes in the virome were suggested to contribute to intestinal inflammation and bacterial imbalance or dysbiosis^36^. Herpesvirus latency was suggested to confer symbiotic protection from bacterial infection^37^. In a mouse model of latent mouse CMV infection, resistance to *Listeria monocytogenes* and *Yersinia pestis* was observed, suggesting that latency may upregulate the basal activation state of innate immunity against subsequent bacterial infection. In a recent study in mice NOD2 deficiency was associated with changes in the structure of microbial communities that are detrimental to the host and lead to colitis and colitis-associated carcinogenesis, suggesting a role for NOD2 in modulating gut bacterial communities^38^. Based on our *in vitro* data the effect may be bi-directional, in that MDP-containing microbial populations may affect HCMV replication. It is possible that bacterial decolonization may result in a more conducive environment for HCMV replication. In line with our theory, a study that used HFFs and ectocervical tissue revealed that defined TLR ligands inhibit HCMV via IFN-β. The authors suggested that different types of flora in the female genital tract can influence virus replication and that reactivation and shedding of HCMV in the genital tract may be determined by alterations in the normal flora, which results from underlying conditions such as bacterial vaginosis or sexually transmitted diseases^39^. Future animal studies will be needed to confirm these findings.

In summary, our study presents the unique activities of bacterial MDP on HCMV suppression via NOD2 activation either following infection or in the setting of persistent NOD2 activation. The latter may represent changes in bacterial colonization which commonly occurs *in vivo*. Intestinal expression of NOD2 has been suggested to be regulated by the microbiota, since germfree mice had lower NOD2 expression that was restored upon colonization with commensal bacteria^40^. Our data may support a model for bacteria-HCMV cross talk providing a protective antiviral environment.

## Additional Information

**How to cite this article**: Kapoor, A. *et al*. Bacterial Muramyl Dipeptide (MDP) Restricts Human Cytomegalovirus Replication via an IFN-β-Dependent Pathway. *Sci. Rep*. **6**, 20295; doi: 10.1038/srep20295 (2016).

## Supplementary Material

Supplementary Information
