# Supplementary material for: Bacterial Muramyl Dipeptide (MDP) Restricts Human Cytomegalovirus Replication via an IFN-β-Dependent Pathway
Source: Sci Rep. 2016 Feb 2;6:20295. doi: 10.1038/srep20295 (PMC4735818; doi:10.1038/srep20295)
Supplement: Supplementary Information [file srep20295-s1.pdf]

Bacterial Muramyl Dipeptide (MDP) Restricts Human Cytomegalovirus Replication via an IFN-β-Dependent Pathway

**Arun Kapoor, Yi-Hsin Fan, Ravit Arav-Boger***

Department of Pediatrics, Division of Infectious Diseases, Johns Hopkins University School of Medicine, Baltimore, Maryland 21287, USA, Email: akapoor7@jhmi.edu, yfan17@jhmi.edu.

*Corresponding author: Ravit Arav-Boger, MD, 200 N. Wolfe St. /3153, Baltimore, MD, 21287, Phone: 410-614-3917 Fax: 410-614-1491, Email: boger@jhmi.edu

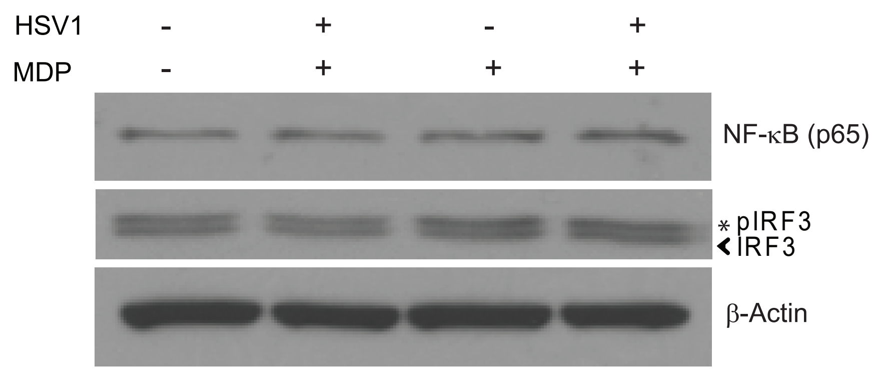


**Figure S1: MDP treatment does not affect NOD2 downstream signaling in HSV1 infected cells**. Cells were infected with HSV1 (MOI 0.1), and treated with MDP for 6 h. Expression of NF-κB and IRF3 was determined by Western blot. β-actin was used as loading control. Western blot data are from a representative experiment of three independent experiments.

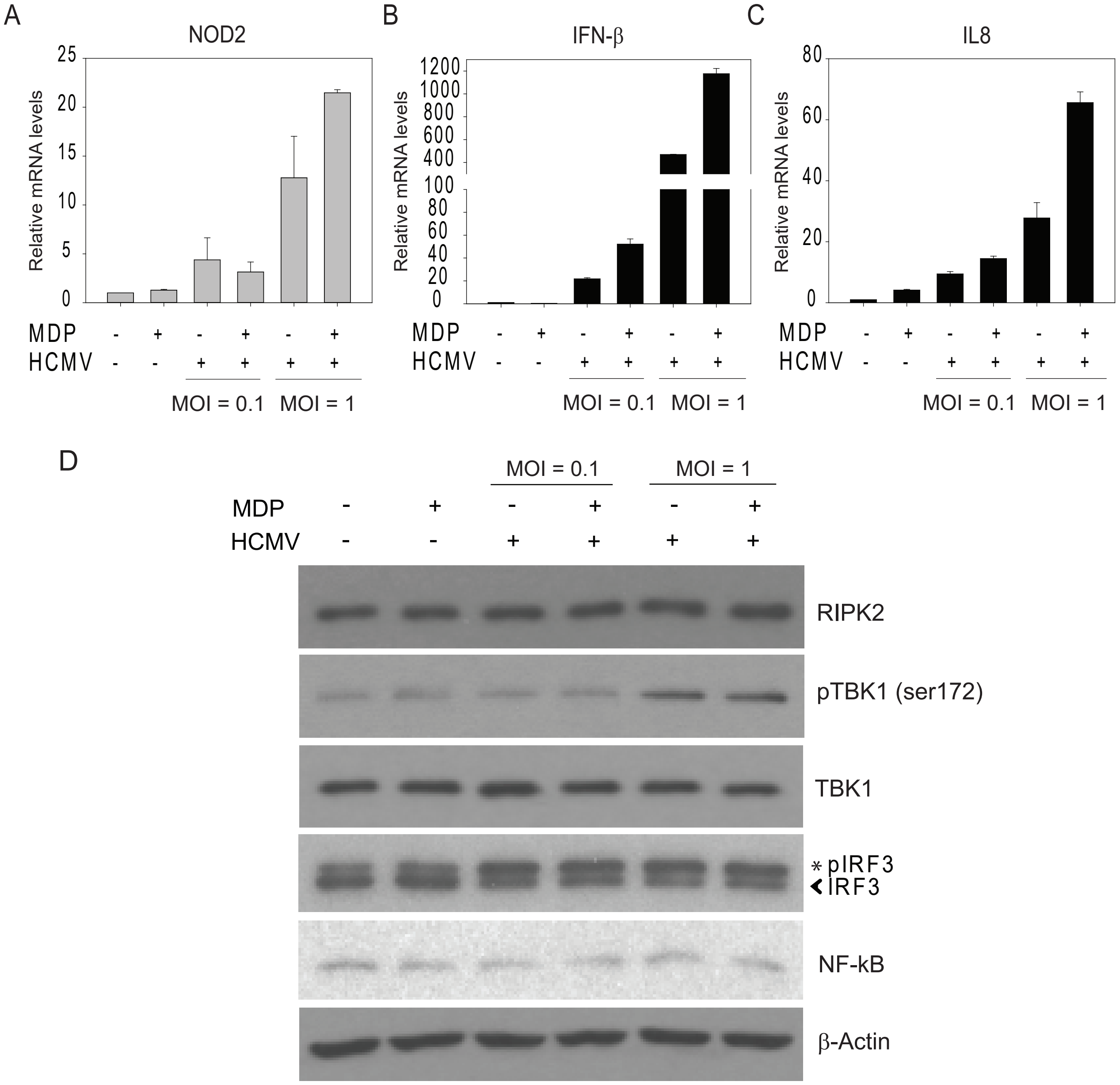


**Fig. S2**: **Expression of antiviral and inflammatory cytokines at 4 hpi and MDP treatment. A, B, C.** Cells were infected with HCMV (MOI 0.1 or 1) and after 90 minutes treated with MDP. The expression of NOD2 (A), IFN-β (B), and IL8 (C) mRNA was measured at 4 hpi. Changes in IL8 and IFN-β expression were not significant in infected MDP treated HFFs. **D.** The expression of proteins downstream of NOD2 was detected by Western blot. Representative data from two-independent experiments (for A-D) are shown.

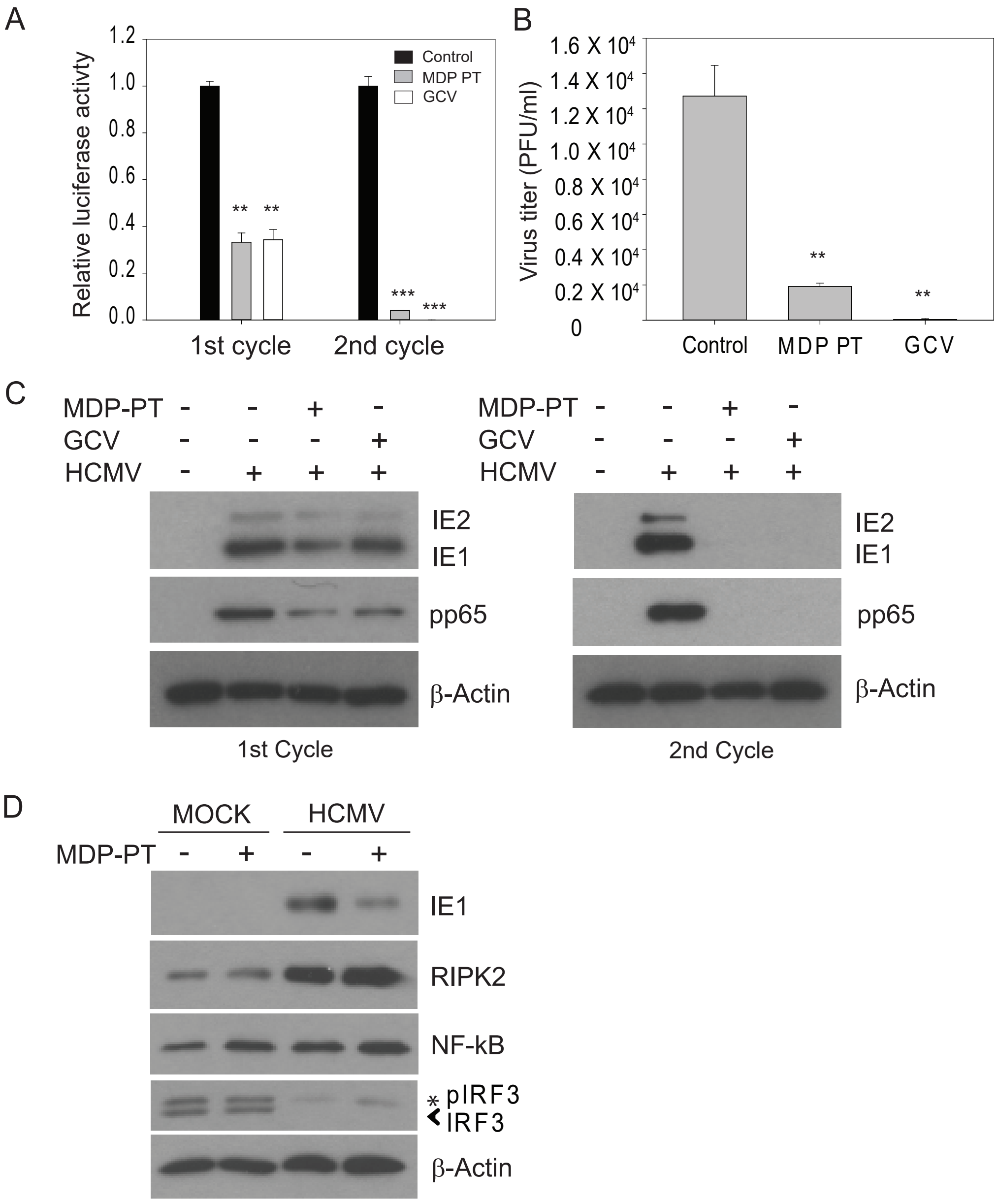


**Fig. S3**: **Effect of MDP pretreatment on HCMV replication (purified virus). A.** Cells were pretreated with MDP for 72 h followed by infection with a purified pp28 luciferase-recombinant HCMV (MOI 1). Luciferase activity was measured in cell lysates at 96 hpi (1st cycle). Supernatants were collected at 96 hpi, and used for infection of fresh cells (2nd cycle) and luciferase activity was measured at 72 hpi. Luciferase data are mean ± SD from triplicate measurements of a representative experiment (**p< 0.01, ***p<0.001, one-way ANOVA test). **B.** Supernatants from A were collected to infect fresh cells and virus titer was determined by plaque assay after 10 days. **C.** Levels of IE1/2, pp65 and β-actin in cell lysates from 1st and 2nd cycle (**A**) were determined by Western blot. **D.** Cells were pretreated with MDP for 72 h followed by infection with a purified pp28 luciferase-recombinant HCMV (MOI 1) for 24 h. The expression of IE1, and NOD2 downstream signaling proteins was detected in total cell lysates by Western blot.
